# The Potential Role of Epigenetic Modifications on Different Facets in the Periodontal Pathogenesis

**DOI:** 10.3390/genes14061202

**Published:** 2023-05-30

**Authors:** Samuel Laberge, Daniel Akoum, Piotr Wlodarczyk, Jean-Daniel Massé, Dominique Fournier, Abdelhabib Semlali

**Affiliations:** 1 Groupe de Recherche en Écologie Buccale, Faculté de Médecine Dentaire, Université Laval, Québec, QC G1V 0A6, Canada; samuel.laberge.3@ulaval.ca (S.L.); daniel.akoum.1@ulaval.ca (D.A.); piotr.wlodarczyk.1@ulaval.ca (P.W.); jean-daniel.masse.1@ulaval.ca (J.-D.M.); 2 ERDOMO LLP, England, and Wales, Whitley Bay NE25 8XT, UK

**Keywords:** smoking, nucleotide excision repair, XPA, XPC, single-nucleotide polymorphism, Taq man genotyping

## Abstract

Periodontitis is a chronic inflammatory disease that affects the supporting structures of teeth. In the literature, the association between the pathogenicity of bacteria and environmental factors in this regard have been extensively examined. In the present study, we will shed light on the potential role that epigenetic change can play on different facets of its process, more particularly the modifications concerning the genes involved in inflammation, defense, and immune systems. Since the 1960s, the role of genetic variants in the onset and severity of periodontal disease has been widely demonstrated. These make some people more susceptible to developing it than others. It has been documented that the wide variation in its frequency for various racial and ethnic populations is due primarily to the complex interplay among genetic factors with those affecting the environment and the demography. In molecular biology, epigenetic modifications are defined as any change in the promoter for the CpG islands, in the structure of the histone protein, as well as post-translational regulation by microRNAs (miRNAs), being known to contribute to the alteration in gene expression for complex multifactorial diseases such as periodontitis. The key role of epigenetic modification is to understand the mechanism involved in the gene-environment interaction, and the development of periodontitis is now the subject of more and more studies that attempt to identify which factors are stimulating it, but also affect the reduced response to therapy.

## 1. Historical Evidence in the Association between Genetics and Periodontal Diseases

**Figure 1:**
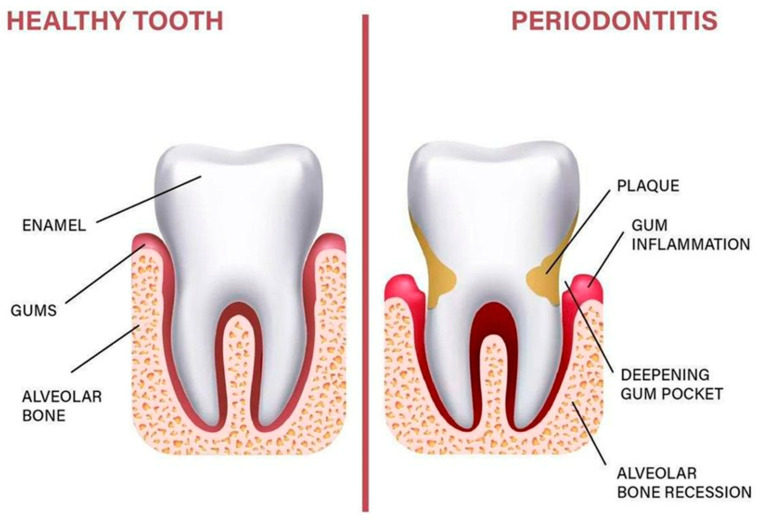
Anatomy of the periodontium.

Periodontitis (PD) is a chronic inflammatory disease of microbial origin. Its primary etiological factor is dental plaque. In fact, the bacteria in plaque contribute to the establishment of an inflammatory environment by stimulating the host response. In a person who does not develop the disease, the inflammation resolves itself and allows the maintenance of gingival health. In others, it persists and becomes chronic to afterward cause a reduction in periodontal support that can be observed by clinical attachment loss and radiographic bone loss [1] (Figure 1).

There is a lot of evidence reporting that PD is a multifactorial chronic inflammatory condition closely related to genetic modifications. A demonstration of the genetic contribution to its onset and severity was recognized as early as the 1960s. The first piece was pointing out a higher risk of developing PD when a parent or sibling was affected, thus a lot of work on animal models and humans investigated its heritability [2,3]. Based on twin and family research, and more recently on genome-wide association studies (GWAS) [4], the heritability of PD was estimated between 30% and 50% [5,6]. From these findings, the periodontal health status was very similar in identical twins, whether they are raised in the same environment or not. Moreover, it was observed that patients who suffer from periodontitis have one of their parents’ trends of losing teeth at a relatively young age [5,6]. Using this information, we conclude that the capacity of an individual to protect/educate himself against gum problems is partly hereditary. Indeed, PD susceptibility genes have been identified, and this trait can be passed on from parents to children. This evidence can help elaborate an assessment of the person’s predisposition to disease by issuing instructions on prevention and treatment for each population. By gathering current discoveries on the correlation between genetic and periodontitis development, this could lead to a better identification of the susceptibility to disease and improve its clinical aspect by creating targeted interventions based on this same susceptibility. The aim of this review is to explore the role of genetic and epigenetic mechanisms in the patient’s susceptibility being characterized and to discuss its practical integration in the clinical field.

**Figure 2:**
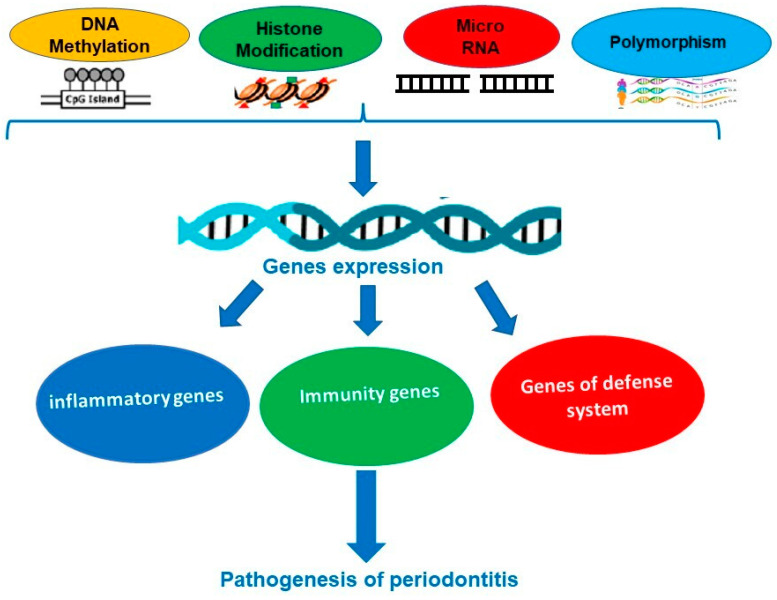
Some epigenetic mechanisms involved in periodontal diseases.

**Evidence of epigenetic modifications (polymorphism, gene expression, and microRNA) and periodontal disease development.** With the new technology in genetic analysis and rapid advances in epidemiology, the crucial role of epigenetic modification involved in the onset and progression of PD has become increasingly important, both in diagnostic tests and in the response to proposed treatments [7,8,9] (Figure 2). Do gene polymorphisms affect the purpose of treating PD whether a surgical or a nonsurgical approach is used? This was mainly investigated by Chatzopoulos et al. in recent publications. In their paper from 2017, they concluded that IL-6 −572 G/C and IL-10 −592 C/A alone or in combination have no effect on the nonsurgical therapy received by Caucasian people being chronically ill [10]. Later, this finding was validated by a 3-year study [11]. It seems that no data are available for patients treated surgically. Using a conventional method that targets the bacteria or the pathogens, it is limited if the disease is going to recur or there is antibiotic resistance. New treatment strategies are highly advisable, especially if they looked at the periodontium chronically inflamed, this being based on an innate immune mechanism of the host response. Lavu et al. concluded that insights on how histones are modified, DNA is methylated, and microRNAs are regulated will increase understanding of the molecular background behind chronic inflammatory conditions [12]. To validate the influence of the epigenome, existing wide analyses were published on this subject [13,14].

**Figure 3:**
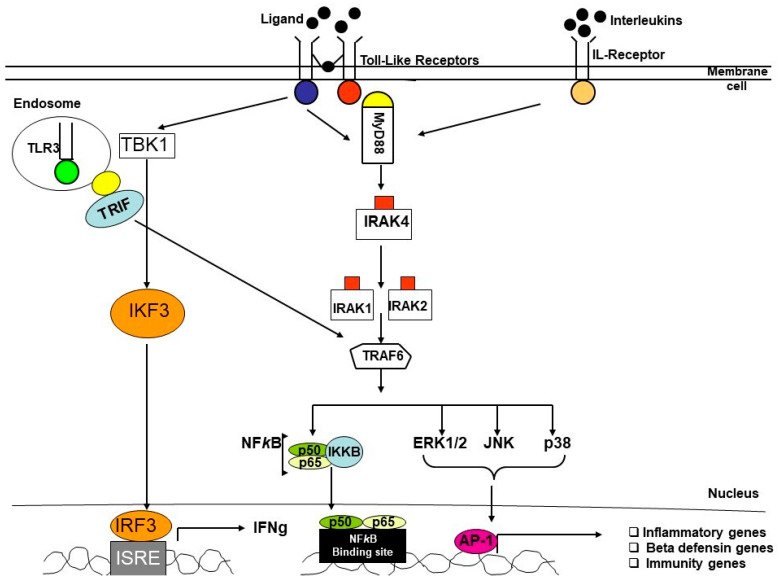
Simplified diagram of *IL-R* and *TLRs* pathways in inflammatory diseases.

As the inflammatory, immune and defense systems are the main elements that are playing a critical role in the pathogenesis of periodontitis, any changes in the genes may influence the responses to inflammation and immunity in the disease. Mutations in single genes involved in many disorders, such as CHS (Chediak-Higashi syndrome), appear to be associated with increased susceptibility to PD in patients suffering from this condition [15]. It is also possible to determine periodontitis susceptibility with a single-locus variant. Therefore, several studies have focused on identifying gene expression and genetic polymorphisms in inflammatory and immune proteins, in several features of PD, in various populations. It was observed that allelic variants at multiple genetic loci probably influence its susceptibility [16]. Many reports have demonstrated that pro-inflammatory cytokines play a significant role in the pathogenesis of PD and single-nucleotide polymorphisms (SNPs) in their genes were strongly linked to the risk for the disease and its severity [17,18,19]. Defensins are antimicrobial peptides, crucial components of innate immunity, and extensively present in our oral environment. Numerous studies have reported the association between gene expression and polymorphism of β-defensins (*DEFB)* with the risk of severe PD [20,21]. As the primary response to pathogens in our innate immune system is mainly triggered by Toll-like receptors (*TLRs*), a lot of research has noted that polymorphisms in *TLRs* genes were associated with PD [22,23,24] (Figure 3).

It was described that miRNAs have highly dynamic characteristics to regulate (activate or silence) gene expression in many genetic targets by inhibiting the transcription and translation or by linking with chromatin-remodeling proteins. They are small, single-stranded, and have non-coding RNA sequences. In fact, miRNAs seem to be good molecules to determine the stages of periodontal disease because they tend to have high stability in various biological samples and their dysregulation appears to be associated with different pathologies such as periodontitis [25]. This abnormality or impairment can be induced by bacteria, more precisely bacterial components found in plaque surrounding teeth [25]. Aberrant expression of miRNAs leads to dysregulated cellular responses, such as adaptive and innate immunity, which is a contributing factor in chronic inflammatory diseases [25]. In the case of PD, the speed and severity of its progression are associated with a balanced oral microbiota, but also the immune-inflammatory responses, and it is fair to say that, by extension, they are also associated with microRNA expression [25]. In the current review, we investigate the potential role of epigenetic modification in inflammatory and innate immunity genes, as well as those involved in the defense system, in the pathogenesis of PD. To better understand the genetic modifications in PD is to create a formidable tool for prevention that could allow us to identify individuals at risk of developing periodontitis before they present symptoms of the disease, and therefore to try to prevent its appearance by regular check-ups or a particular attention to oral hygiene. However, PD is a multifactorial disease, in which case an appropriate diagnosis cannot only be provided by genetic screening and must be combined with analysis of the candidate’s environmental factors. Subsequently, in affected patients, genetic tests can inform the determination of the prognosis for the remaining teeth and steer the choice of a special therapy. While there is no clear evidence yet, genetic variants may influence their response to treatment and, in that case, individuals may benefit from another, more intensive therapeutic option, for example, more frequent recalls, different health approaches and more. Finally, the possible identification of genes associated with periodontitis could help in understanding these diseases and thus in the development of new biological treatments.

## 2. Association between Epigenetic Modifications in Host Immune Response Genes and Susceptibility to Periodontal Disease

**Figure 4:**
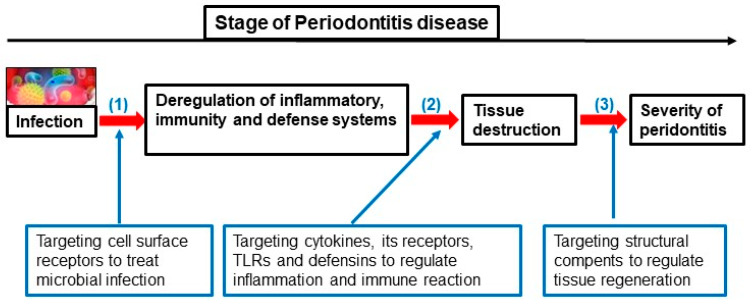
Different stages of periodontitis evolution and targeting genes for their treatment strategy.

Periodontitis’ onset is based on multiple etiologies and mechanisms and is affected by pathogenic bacteria, environmental and genetic factors. This section of the present review summarizes the changes in certain immune genes associated with the development and control of this disease (Figure 4). The progression of oral disease is strongly related to the host immune response. This is guided by key elements from the innate and adaptive immunity of the host, and cells that intervene on the evolution of periodontitis by producing a series of cytokines, chemokines, growth factors and by expressing specific receptors [26]. All these effectors are interrelated in their action to create a unique and highly coordinated immune response by the host [26]. Most particularly, cytokines are essential to periodontal tissue homeostasis and to the regulation of its inflammation after the infiltration of pathogens, such as *P. gingivalis*, *T. denticola* and *T. forsythia* [27]. Recent science proves that SNPs in cytokines with the association of receptor-encoding genes can determine the severity of periodontitis [28,29,30,31]. Based on the cytokine network, a lot of research tends to create clinical therapies centered on these important effector molecules of the immune system, on the genetic regulation and epigenetic control of their expression [27]. Ultimately, the study of immune response genes and their susceptibility to genetic and epigenetic alterations could identify the vulnerability of a patient to develop periodontitis. Making biomarkers that are present in periodontal tissues could be a great help in the diagnosis of the disease.

As with all epithelial tissues of the human body, the periodontium is exposed to a specific microbiota and other stimuli that boost the consistent immune surveillance and tolerance [32]. When the pathogenicity of the oral microbiota achieves a particular level of potential toxicity for the tissue, the innate immune system activates a much more coordinated and elevated inflammatory-related response [27]. If it is over-exaggerated or non-compensated, it can eventually lead to undesirable outcomes associated with periodontitis, such as bone resorption and tooth loss [32]. When the immune response is undertaken against pathogens, an important infiltration of cells occurs, which can be distinguished in three major groups: mononuclear phagocytes (MNPs), antigen-presenting cells (APCs) and specific T helper lymphocytes, such as Th1 and Th17 [27]. These are effectors of the innate immune response, and they are its first actors to produce cytokines [27]. Their secretion is activated by a unique pattern-recognition receptor. In its early stage, mainly interleukin-1 (*IL-1*), the *IL-6* family cytokines, and tumor necrosis factor (TNF) are released [27]. These are all essential pro-inflammatory proteins stimulating the setting of an immune response as a way for defending the organism [33]. The innate immune system serves as its first line of defense and triggers an immediate response to injury, infection, or pathogens [34]. It is a somewhat general and rapid reaction delivering immune cells at the site where the cause is through the release of some cytokines (*TNF*, *PGE2*, *IL-1β*, and more) [35]. This response also initiates the process of phagocytosis and activates the complement and adaptive immune systems [35]. On the other hand, the latter is specifically tailored to certain pathogens and is mediated by T and B cells. It takes more time to activate and generate specialized cells with distinct functions such as recognizing antigens (self from non-self), eliminating pathogens, and creating immune memory [34]. The interaction between innate and adaptive immunity is complex, both are essential in maintaining health and homeostasis, they consist of humoral and cellular immune responses, which include antibody production and defense mechanisms [36]. The inflammation triggered by injury, infection or pathogens is usually self-limiting and leads to tissue repair. However, if this response is altered, it can cause chronic inflammation and autoimmune disorders [37]. This is thought to be a major issue of health problems, including immune-mediated inflammatory diseases such as periodontitis [34]. It is a highly prevalent, if not the most prevalent, form of inflammation in the tooth and its periodontium. This disease is initiated by the dysbiosis of the oral microbiome and quickly evolves into an inflammatory and immune response [36]. When maintaining a constant aggression of the tissue by pathogens, this reaction shifts from an innate to an adaptive one. This is characterized by the contribution of many different immune cells [38]. The progression of periodontitis is very much affected by the response of the host’s immune system. The effectors of innate immunity, such as macrophages, can defend the host against invading microbes; they produce a wide range of cytokines, which in turn trigger an inflammation cascade where its intensity is targeted directly by their concentration [39]. This interaction between the immune effector cells and the inflammatory response to an invasion, such as that observed in periodontitis, is highly upheld by the gene expression regulated by the host’s DNA sequence. Additionally, the regulation in this sequence is often maintained by an epigenetic phenomenon known as DNA methylation, which in turn leads to an organized expression of the host’s immune response while developing periodontitis. DNA methylation is when DNA methyltransferases (DNMTs) catalyze the addition of a methyl group to the sequence, mainly at the C5 position of cytosine in the context of cytosine-phosphate-guanine (CpG) dinucleotides. When an abnormal expression of DNMTs occurs, this can lead to pathological conditions such as periodontitis. Their role to maintain the methylation of CpG sites in the DNA sequence is a regulatory mechanism in which methylated transposable elements are silenced for the benefits of the host. It is when the pattern is changed that the latter is susceptible to numerous infections and has a disrupted immune and inflammatory response [39]. The patients with these altered DNA methylation patterns could be more vulnerable to develop periodontitis, according to Larsson and al. [40]. From Sima and Glogauer [41]. It was reported that in DNA the regulation of macrophage activation and cytokine signaling cascades were associated with DNA methylation patterns. According to Luo et al. [42], we can elaborate on the relevance of this altered methylation to the pathogenesis of periodontitis, by accepting the fact that macrophages are themselves regulated by different patterns. Nonetheless, by knowing the potential correlation with macrophage-dominated immune response and DNA methylation alteration, it can also lead to an opportunity for clinically using this specific methylation status as a biomarker to treat periodontitis [39].

Epigenetics not only includes the contribution of DNA methylation, but it also comprises chromatin remodeling, histone modification, and microRNAs (miRNAs). They have an important role to play in the regulation of biological and physiological factors through the induction of gene silencing. In fact, miRNAs, when over- or under-expressed, can lead to the development of pathological processes such as periodontitis [43]. The pathway for gene silencing in the 22 nucleotides that make up the miRNAs consists of specific binding to their 5′ end, called the seed sequence, and to the 3′-untranslated regions (3′-UTRs) of the targeted mRNAs [43]. The miRNAs, by regulating the post-transcriptional gene expression, influence many biological processes in human cells that depend on DNA transcription and protein synthesis. From cell proliferation and embryonic development to neoplastic progression and cardiovascular diseases, miRNAs have a wide range of actions on the body maintenance. A single miRNA can target multiple genes [44]. This capacity to be involved in the regulation of abundant biological mechanisms point to a possible key role of miRNAs in the periodontal pathogenesis. Recent studies have identified some specific miRNAs as potential causal agents of periodontitis-associated processes, such as miR-146a/b, miR-155, miR-200, miR-203, and miR-223 [43,45,46]. More interestingly, instead of simply determining which miRNAs are over- or under-expressed in the periodontium, an improved understanding of the miRNA effects on immune response pathways could provide a better overview of how those different miRNAs impact the periodontitis development. While the immune system is very complex and diverse, a direct contribution of miRNAs has been reported in autoimmune disease, as observed in the case of lupus patients. When increased, miR-148a is targeting three control molecules in autoreactive B cells: an autoimmune suppressor (Gadd45α), a tumor suppressor (*PTEN*) and a pro-apoptotic protein (*Bim*) [47]. The inflammatory phase of periodontitis is conjointly modulated to the immune response by the complex cytokine network, as briefly described previously. More miRNAs have an indirect impact on it by targeting central receptors and protein-coding genes involved in periodontal inflammation. Four miRNAs have been demonstrated to modulate the innate immune response associated with gingival epithelial cells in periodontitis patients. miR-105 targets Toll-like receptor 2 (*TLR2*) on gingival keratinocytes, which results in two major pro-inflammatory cytokines, *IL-6* and TNFα that are produced in an intensified way [48]. Moreover, from the gingival tissue in the periodontium, it has been shown that miR-203 expression is enhanced by the presence of *P. gingivalis*, one of the main pathogens in periodontitis, where an over-expressed miRNA leads to an activated STAT3 [49]. An active STAT3 arises from the suppressor of the cytokine signaling 2 (SOCS2) being inhibited, due to the action of miR-203 [49]. As a reminder, we previously discussed the impact of STAT3 activation on Th17 cell overproduction, ultimately resulting in an increased inflammatory response. Another miRNA interesting for its implication in the immune reaction to periodontitis is miR-146. In fact, miR-146 binds to IL-1 receptor-associated kinase 1 mRNA, thus blocking the TLR signaling pathway in the infection with periodontopathogens [50]. We cannot consider separately the strong interrelationship between the inflammatory and immune responses, but better comprehension of the various cytokine- and receptor-related genes targeted by miRNAs allow for the identification of which immune cells are upregulated or downregulated depending on the miRNA expression pattern. According to Irwandi and Vacharaska (2016), more than 60 miRNAs have been recognized to be actively involved in the immune/inflammatory response as well as in the gingival homeostasis.

## 3. The Role of Epigenetic Modifications on Inflammation Genes and Susceptibility to Periodontal Disease Development

Chronic periodontitis (CP) is a set of inflammatory conditions affecting the gum tissues, and consequently the immune system’s response is a key element in the exploration of the pathways resulting in this disease. To keep homeostasis under control, there must be a balance between pro- and anti-inflammatory responses, which is guided by local bacteria [51]. When changes occur in the oral cavity, such as diet, aging, or familiar predisposition, the level of aggressive bacteria (*P. gingivalis*, *T. forsythia*, *T. denticola*, etc.) increases compared to the protective ones, a phenomenon called oral dysbiosis, which eventually leads to the inflammatory environment in CP [51]. This inflammation is, in other words, a phenotype clinically observable in a patient’s oral cavity [51]. For each, one or many genes from the genotype, the genetic constitution of a human being, must be expressed or suppressed. Epigenetics, guided by the individual’s behavior or environment, can modify gene expression without altering the DNA sequence itself, and thus can influence the inflammatory process by repressing or overstimulating genes [51]. Studies have shown that epigenetic events contribute to the development of conditions, such as airway inflammation and severe systemic inflammation. Periodontitis (PD) could be another one of these diseases [51]. Oral hygiene, smoking, lack of nutrition and exercise, and use of drugs are environmental factors influencing epigenetic patterns that can result in infections such as periodontal diseases [51]. Many genes are well-documented and stand out from others when it comes to understanding the impact of epigenetic changes during inflammation leading to PD, which are prostaglandin-endoperoxide synthase 2 (PTGS2), tumor necrosis factor-α (*TNF-α*), and interleukins (*IL-1β* [24], *IL-6* [25], *IL-8* [26] and *IL-10* [27]). In humans, when expressed, the PTGS2 gene encodes for a protein also called cyclooxygenase-2 (COX-2), an enzyme involved in the conversion of arachidonic acid to prostaglandin H2 in the processes of inflammation and pain [28]. On the other hand, it has been reported that COX-2 inhibitors were able to reduce the symptoms in patients suffering from periodontitis. This means that PTGS2 becomes a key recognition gene which, when targeted by an epigenetic mechanism, contributes to dysregulating inflammatory signals [28]. *TNF-α* induces the production of collagenase and prostaglandins by gingival fibroblasts, leading to its major role in tooth-related attachment loss in PD [29]. IL-1β, generated by activated macrophages, is a mediator of the inflammatory response in many ways, including cell proliferation, differentiation, and apoptosis. IL-6 is a pro-inflammatory cytokine-like *TNF-*α and *IL-1β*, but could also play a protective role. Indeed, although IL-6 is involved in recruiting neutrophils for the acute inflammatory response, this protein also inhibits pro-inflammatory cytokines such as TNF-α and IL-1 and activates anti-inflammatory cytokines such as *IL-10*. *IL-8* is, however, a cytokine produced by macrophages, which triggers the chemotaxis of neutrophils and stimulates phagocytosis in infected sites [30]. IL-10, as explained above, has a protective role where it reduces inflammation and the immune response by macrophages with T cells by inhibiting the production of cytokines such as *TNF-α.*

### 3.1. Methylation and Acetylation in Inflammatory Genes Associated with Periodontitis

The most documented process of epigenetics is DNA methylation. This chemical reaction can be described as adding a methyl group at a specific location on the DNA sequence of a gene, where a cytosine is directly followed by a guanine, called the CpG site [31]. The transfer of a methyl group to the cytosine converts it into another form named 5-methylcytosine, and this reaction is catalyzed by enzymes known as DNA methyltransferases [31]. Therefore, this mechanism leads to repression of genes by contributing to the prevention of transcription factors bound to DNA. DNA methylation modifies cell functions, including DNA repair, regulation of cellular proliferation, and inflammatory gene expression, which could result in defects in the phenotype of proliferating cells, such as epithelial cells, fibroblasts, and bone-forming cells [52]. In the progression of periodontitis, the purge of those cells by apoptosis cannot be reverted, which contributes to the tooth loss. A significant difference has been reported in the level of DNA methylation between healthy patients and people suffering from an aggressive periodontal disease and having chronic conditions. Moreover, the constant presence of *P. gingivalis* in epithelial cells induces DNA methylation (in vitro) [53]. In cases of CP, the methylation patterns of IL-8 and PTGS2 genes are altered when compared to healthy subjects [54,55].

The acetylation of histone H4 is another epigenetic mechanism that can be associated with the development of PD [56]. This reaction is characterized by the process of adding an acetyl group to lysine residues (amino acids) located at the transcription start site of histones (N-terminal tail), a protein structure around which DNA winds itself to form nucleosomes. Acetylating amino acids can in fact lead to increased expression of genes coding for TNF and IL-1β and, on the other hand, suppression of histone acetylation has a positive effect in preventing alveolar bone loss.

### 3.2. miRNA Dysregulation and Modulation of the Inflammatory Genes’ Expression

MicroRNA (miRNA) is a small, non-coding RNA molecule that plays an important role in gene regulation. They are involved in a wide range of biological processes, including inflammation. Dysregulation of miRNA expression has been linked to several diseases, such as chronic periodontitis. Several miRNAs have been identified as potential mediators of the inflammatory response in PD. For example, miRNA-21 has been shown to promote inflammation by inhibiting the expression of many anti-inflammatory proteins, such as programmed cell death protein 4 (PDCD4) and transforming growth factor, β-induced (TGFβ-1). Additionally, it has been demonstrated that mi-RNA-146a regulates inflammation by targeting interleukin-1 β (IL-1β), a pro-inflammatory cytokine. Dysregulation of miRNA expression in periodontitis is thought to be mediated by several factors, including oxidative stress and stress-activated signaling pathways [57,58]. Oxidative stress, which is an imbalance between the production of reactive oxygen species (ROS) and the availability of antioxidant defenses, has been shown to affect the expression of miRNAs in periodontal tissues [59]. There is evidence suggesting that miRNAs can be used as diagnostic and prognostic markers in periodontitis. For example, the expression levels of miRNA-21 have been shown to be increased in the gingival crevicular fluid of patients with PD, and their concentration has been correlated with the severity of the disease [60]. In addition, it has been demonstrated that of miRNA-146a is decreased in the gingival crevicular fluid of periodontal patients, and their levels were related to the disease progression [61].

### 3.3. Single-Nucleotide Polymorphisms of Inflammatory Genes and Periodontal Disease Progression

In addition to epigenetics through DNA, another phenomenon relevant to explore is the imbalance between pro- and anti-inflammatory responses in the oral cavity, leading to the development of periodontitis, which is single-nucleotide polymorphism (SNP). SNPs can be described as the substitution of a single nucleotide at a precise location in a gene, which can lead to an abnormal expression and eventually modifies its phenotype. In chronic periodontitis, SNPs in the promoter region of the IL-10 gene can be a determinant variable to understand the impairment in its expression, resulting in a deficient aggravated anti-inflammatory response with respect to this disease [62,63,64]. Additionally, polymorphisms have been observed in genes cited above, as TNF-α [65] and IL-1β [66,67], in patients with CP, which influence their expressions, and contribute to the disproportion of pro- and anti-inflammatory factors. Thus, single-nucleotide polymorphisms can be useful to appreciate the role of oral dysbiosis leading to chronic inflammation in periodontitis. It could help to have a better disease diagnosis and severity assessment with the purpose of improving therapeutical practices.

## 4. The Role of Epigenetic Modifications in Genes’ Defense Mechanisms and Susceptibility to Periodontal Disease

### 4.1. Changes in Antimicrobial Peptides

Chemical modifications in DNA due to epigenetics, polymorphisms or mutations may alter cellular functions and defenses by affecting the properties of gene expression. Some bacteria found in the mouth are also known to influence these functions through their toxic activity [68]. The oral mucosal surface must differentiate commensal microorganisms from pathogens to properly coordinate inflammatory responses. Constant inflammation in the periodontium due to microbial biofilm results in natural cell death and turnover, causing potential epigenetic changes during the regeneration process and, therefore, may affect its phenotype and defense response [69]. While good hygiene leads to better oral health and lowers the inflammatory burden, habits that promote cellular modification such as smoking, poor nutrition, lack of exercise and use of drugs greatly influence epigenetic patterns in this process and inflammatory reactions [70]. Some areas of the mouth are more susceptible to periodontal disease from patient to patient because of high turnover rates and remain vulnerable even after treatment because of their prolonged inflammatory response, which may have caused epigenetic alterations. There are no more questions to be asked in saying it is the magnitude of this cellular response rather than the bacterial infection that causes destructive periodontal disease [71]. Therefore, knowing the impact of genetics (and its potential alteration) in the response, research in this area is a key to fully understand periodontitis. Among the multiple local mechanisms, to protect the organism from pathogens, oral cells have antimicrobial peptides (AMPs), produced by polymorphonuclear leukocytes (PMNs) in the gingival crevicular fluid (GCF), human gingival fibroblasts (HGFs) or gingival epithelial cells (GECs) through IL-8 stimulation, and directly by GECs, the most well-studied being defensins, cathelicidins and histatins [72,73]. In addition to AMPs, there are several other types of antimicrobial molecules made by cells in the periodontal pocket that play a role in the defense against infections, the best studied being lactoferrin and lysozyme [74].

Human β-defensins (hBDs) are cationic AMPs produced in part by gingival epithelial cells. They are an essential key factor in local immediate innate immune response characterized by their ability to form pores in the bacterial membrane, leading to cell death [75,76]. Defensins have broad-spectrum antimicrobial activity and have been shown to be effective against Gram-negatives, viral and fungal infection [75]. hBD-1-3 are known to be expressed in the human oral cavity [77]. While hBD-1 is constitutively released, hBD-2 and hBD-3 secretions depend on local infection and inflammation [78]. These AMPs are working by chemoattraction of dendritic and T cells, and macrophages to the site [79].

Cathelicidin LL-37 is also a cationic peptide that is produced by macrophages and gingival epithelial cells. It inhibits a wide range of microorganisms and has proven to be effective against a variety of bacteria, including *P*. *gingivalis*, which is a major pathogen in periodontitis [80]. It is known as well to neutralize the lipopolysaccharide from Gram-negatives. LL-37 has also been shown to have immunomodulatory effects and to enhance the migration and activation of neutrophils [81].

Histatins (HTNs) are cationic AMPs that are produced by salivary gland epithelial cells with a broad-spectrum antimicrobial activity. They are effective against a variety of oral pathogens, including *C*. *albicans* and *P*. *gingivalis* [82]. Histatins have indeed been shown to exert anti-inflammatory effects and to inhibit the production of pro-inflammatory cytokines against such bacteria. It was also demonstrated that these proteins are inhibiting bacterial coaggregation and the activity of their proteases in laboratory studies, suggesting potential applications in periodontal treatment.

Lactoferrin (LF) is an iron-binding protein that is formed at mucosal surfaces and has antimicrobial activity against bacteria, viruses and fungi. It has been shown to be effective against a variety of bacteria, being present on all these surfaces of the body [83].

Lyzozyme (LYZ) is an enzyme that comes from mucosal epithelia and possesses antimicrobial activity against Gram-positives. It can be in saliva and in the inflammatory exudate found in the gums (gingival crevicular fluid). It mainly functions by breaking down the cell walls of Gram-positive bacteria by hydrolyzing peptidoglycans [84].

### 4.2. Imbalance Rather Than under/over Production of Antimicrobial Peptide and PD

In addition to the physical barrier constituted by the epithelium, AMPs secreted by the so-called epithelial cells are an important component in local defense mechanisms that occur early against periodontal pathogens. AMPs having immunomodulatory properties, meaning they can regulate the gene expression to modify its response, are of great interest concerning this study. They also provide a synergistic effect, which significantly increases their effectiveness [85]. For example, it has been shown that cathelicidin (LL-37) and human β-defensin-3 (hBD-3) can combine to further reduce the progression of inflammation by diminishing inflammatory cytokine production in gingival cells [86]. Any modifications in the regulations of secreted AMPs can lead to biological disorders, such as in patients suffering from Crohn’s disease, where an imbalanced hBD-1 release causes abnormal inflammation [87]. This could suggest that AMPs greatly help balance the mucosal immune system frequently exposed to pathogens, such as the pathway from the mouth to the stomach. Indeed, it has been reported that an imbalance in AMPs could be observed in sites affected by periodontitis [85]. For example, it has been found that LL-37 concentration in the GCF increased for patients who are developing it. In this population, AMPs may be expressed at higher levels to fight the inflammation and bacterial infection present in the gums, but it is not clear whether this is the cause or the outcome of the disease. It should be noted though that their levels in gingival fluid could be influenced by systemic inflammation [84]. When analyzing mRNA expression using in situ hybridization, increased levels of transcripts were found for hBD-2 in samples from gingivitis- and periodontitis-affected patients compared to healthy ones. Transcripts for hBD-3 were higher as well in periodontal samples. Interestingly, the expression of hBD-1 seemed to only change when comparing those from aggressive and chronic periodontitis, being greater in the latter one [88]. It is also worth pointing out that some risk factors have shown they can modify AMP expression levels. While diabetic patients presented overall higher concentrations of *hBD-1* and *hBD-3* in their GCF, those with both diabetes and periodontitis overproduced hBD-2 [89]. Smokers, on the other hand, showed a decreased expression of hBDs in their gingival tissue, but an increased level when fighting periodontal infection compared to similar non-smoker patients [90]. A study suggests that smoking could modulate *hBD-2* expression through NF-κB, ERK1/2 as well as MAPK signaling pathways [91]. As for the elderly, lower expression levels of hBD-2 and hBD-3 were observed [92]. Moreover, periodontitis could be linked to disorders such as Kostmann disease, Papillon-Lefèvre and Haim-Munk syndromes that all exhibit significantly lower LL-37 concentrations in affected patients [93]. Consequently, there seems to be a tendency where already infected and at-risk patients would overproduce AMPs, further fueling the existing excessive inflammatory response that comes with periodontitis, while only those with a predisposition appear to underproduce them, leaving their periodontal tissue vulnerable. It just goes to show how periodontitis would indeed be the result of an imbalance in antimicrobials rather than having them in underproduction.

Further supporting this statement, in a recent study, it was noted that DEFA4 (for defensin α 4), LYZ (for lysozyme) and HTN3 (for histatin 3) expression levels showed high sensitivity and specificity regarding their detection and were significantly different between healthy sites and those affected by periodontitis, proving they could be a useful diagnostic indicator in the future. While DEFA4 and HTN3 concentrations were much lower, that of LYZ was higher [85].

### 4.3. AMPs Polymorphisms and PD Development

The AMP gene products can undergo various changes after they are turned into proteins, such as post-translational modifications, and there can also be differences in the genes themselves among individuals. This diversity may help the tissues of the mouth and oral airways resist invasion or infection by the many types of microorganisms that can enter these areas [88]. Alterations in the genetic profile of the peptide are possible as well, such as polymorphisms, often contributing to the disease progression through their impaired expression. For example, in vitro, the presence of a −44 C/G SNP locus in the hBD-1 gene was shown to increase its own mRNA transcript level as well as that of hBD-3. A greater antimicrobial activity was observed in these cells compared to those carrying the common allele [94]. On the other hand, a SNP in the 3′-untranslated region of hBD-1 was associated with periodontal infection [95]. These outcomes are interesting because, as pointed out earlier, higher hBD-1 and hBD-3 expressions were detected in diabetic patients, which are known to be at a heightened risk to develop periodontitis, while the same thing here through the 44 C/G gene polymorphism suggests an increased microbial resistance, though these results are in vitro. In fact, these findings are contradicted by a recent study [96] aiming to verify a link between *DEFB1* and LTF polymorphisms and periodontitis. It was noted that two specific SNPs in the DEFB1 gene, g. 20G > A (rs11362) and g. 44C > G (rs1800972), were significantly related to a greater risk of a serious condition. The 20 G/G genotype was more common among individuals with periodontitis compared to controls, while it was more prevalent for the 44 C/C genotype in healthy subjects in comparison with those having the disease. Additionally, it has been reported that the rs11362 A allele is associated with reduced DEFB1 gene expression while G-20A and C-44G variants have been linked to higher peptide synthesis. Furthermore, for the LTF gene, the p.Lys47Arg SNP was more frequent in people with periodontitis compared to the controls, indicating an increased risk of developing the disease. Moreover, the p.Ala29Thr and p.Lys47Arg SNPs were both associated with a slightly higher risk of periodontitis.

### 4.4. miRNA Influence in hBDs Expression on PD Diseases

Though it is not directly related to periodontitis, a study conducted on Brazilian children in 2017 [97] made an interesting finding by trying to link polymorphisms in *hBD-1* and miRNA202 to a predisposition to caries. Indeed, the data indicated that the variant CC genotype of miRNA202 plays a role in regulating the DEFB1 gene expression in saliva, by binding to its mRNA. This is demonstrated by the fact that children with this genotype had lower concentrations of hBD-1 in their saliva, resulting in more caries susceptibility. This research would then suggest that miRNA202 and its CC phenotype could have a role in the disease progression by down-regulating DEFB1, a gene known to be involved in periodontitis. Based on earlier statements, lower levels of such genes may be synonymous with increased susceptibility to the development of periodontitis. Again, more studies are needed to link miRNA regulation to AMP expression to fully understand their roles in periodontal health.

Regarding AMPs and their genetic variants in general, this section highlighted key evidence that they could be a powerful medical tool in preventing, treating, and assessing disease progression in a cohort. Some peptides being quite easily detected and quantified in saliva, additional research on the subject is needed to define variations that are more specific to AMP levels in periodontitis patients who may benefit from their biomarker potential, as has also been pointed out by Güncü et al. [98].

## 5. Future Prospects Regarding for Interacting New Waysto Diagnose and Treat Periodontitis

### The Emergence of Possible Adjuvants in Clinical Management of Sufferers

Many studies have tested the utility for patient care in relation to existing methods used in other autoimmune or inflammatory diseases. As a matter of fact, clinicians could really benefit from integrating some of these breakthroughs in epigenetic- and anti-inflammatory-related management approaches, which could improve periodontal diagnosis and treatment. Some of these are worth discussing.

Epigenetic modifications previously described could lead to a better diagnosis. For instance, methylation rates in inflammatory genes [99] and miRNA targeting [45] could be used as promising biomarkers for the PD severity. Clinicians could also add to their diagnostic sequence the identification of SNPs evoking greater risks of developing the disease [100].

The similarities in the etiopathogenesis between periodontitis and other immune-inflammatory-related diseases and syndromes made possible the development of new potential treatment methods. In fact, various studies tend to elucidate new targeting therapies regarding precise inflammatory and epigenetic factors of periodontitis development. The use of small-molecule inhibitors of BET proteins, histone deacetylase (HDACs) and DNA methyltransferase (DNMTs) could have a positive impact on periodontal therapy [101]. With further research, even monoclonal antibody medications such as ustekinumab (Stelara^®^), which has been proven to be a good adjuvant in the management of Crohn’s disease by inhibiting the IL-23/IL-17 axis, could be a potential impact to limit the progression of the immune and inflammatory response to PD [34].

Without a doubt, much more research needs to be done on this matter, which could surely produce new and more efficient ways to treat the disease by targeting it at its source rather than doing it only for its symptoms.

## Data Availability

All data generated or analyzed during this study are included in this published article.
